# Number of Positive Lymph Nodes Is Superior to LNR and LODDS for Predicting the Prognosis of Pancreatic Neuroendocrine Neoplasms

**DOI:** 10.3389/fendo.2021.613755

**Published:** 2021-03-05

**Authors:** Bingqiang Gao, Dongkai Zhou, Xiaohui Qian, Yuancong Jiang, Zhenghao Liu, Wang Zhang, Weilin Wang

**Affiliations:** ^1^ Department of Hepatobiliary and Pancreatic Surgery, The Second Affiliated Hospital, Zhejiang University School of Medicine, Hangzhou, China; ^2^ Key Laboratory of Precision Diagnosis and Treatment for Hepatobiliary and Pancreatic Tumor of Zhejiang Province, Hangzhou, China; ^3^ Research Center of Diagnosis and Treatment Technology for Hepatocellular Carcinoma of Zhejiang Province, Hangzhou, China; ^4^ Clinical Medicine Innovation Center of Precision Diagnosis and Treatment for Hepatobiliary and Pancreatic Disease of Zhejiang University, Hangzhou, China; ^5^ Clinical Research Center of Hepatobiliary and Pancreatic Diseases of Zhejiang Province, Hangzhou, China

**Keywords:** pancreatic neuroendocrine neoplasms, log odds of positive lymph nodes, lymph node ratio, number of positive lymph nodes, staging

## Abstract

**Background:**

The American Joint Committee on Cancer (AJCC) staging for pancreatic neuroendocrine neoplasms (PanNENs) based on the number of positive lymph nodes (PLNs) is the most widely accepted nodal staging system. New nodal staging schemes that take both the number of PLNs and the number of examined lymph nodes into consideration have emerged as useful prognostic tools. The aim of the current study was to determine the most effective nodal staging system, among the 8th edition AJCC N staging (or PLN staging), lymph node ratio (LNR), and log odds of positive lymph nodes (LODDS), for predicting the cause-specific survival of patients with PanNENs.

**Methods:**

The clinicopathological and prognostic data of 2,295 patients from the Surveillance, Epidemiology, and End Results (SEER) database, diagnosed with PanNENs between 1988 and 2015, were reviewed retrospectively.

**Results:**

A multivariate analysis identified PLN and LNR staging as independent prognostic factors, but not LODDS. The PLN staging exhibited higher C-index and area under the curve values than those of the LNR and LODDS, indicating better predictive discriminatory capacity. No significant difference in the survival of patients was observed within the same PLN staging subgroup according to the number (high or low) of examined lymph nodes. In contrast, intra-group heterogeneity was seen with use of LNR and LODDS staging, due to overestimation of the risk of insufficient examined lymph nodes, and LODDS failed to stratify patients without lymph nodes metastasis into different risk groups.

**Conclusions:**

The PLN staging is more reliable than LNR and LODDS staging for predicting the cause-specific survival of PanNENs.

## Introduction

Pancreatic neuroendocrine neoplasms (PanNENs) are malignancies arising from the pancreas showing neuroendocrine differentiation (1). Their incidence has significantly increased over the past few decades, probably due to advancements in imaging techniques (2, 3). PanNENs exhibit heterogeneous biological behavior, and include indolent and aggressive types. Based on differences in the mitotic count and Ki-67 labeling index, PanNENs can be divided into well-differentiated pancreatic neuroendocrine tumors (PanNETs) and poorly differentiated pancreatic neuroendocrine carcinomas (PanNECs) (1). In the 8th edition of the American Joint Committee on Cancer (AJCC) Cancer Staging Manual, the nodal staging system for PanNETs is based only on nodal status (negative or positive), while the system for PanNECs is based on the number of positive lymph nodes (PLNs) (4). Although it is challenging to stratify patients into different risk groups due to the rarity and heterogeneity of PanNENs, a unified staging scheme is more attractive in clinical application. Partelli et al. reported that the number of PLNs accurately predicts recurrence not only for PanNECs but also for PanNETs (5). New nodal staging schemes that take both the number of PLNs and the number of examined lymph nodes (or negative lymph nodes) into consideration have emerged as useful prognostic tools. The lymph node ratio (LNR) is defined as the ratio between the number of PLNs and the total number of examined lymph nodes. The LNR is being increasingly recognized as a strong predictor of survival in patients with other pancreatic neoplasms, including pancreatic adenocarcinoma (6) and intraductal papillary mucinous neoplasms (7). Previously, it was reported that an LNR-based staging system is superior to the current 8th edition AJCC staging system for PanNENs (8). Log odds of positive lymph nodes (LODDS) is defined as log (PLNs+0.5)/(examined lymph nodes-PLNs+0.5). Adding 0.5 to the numerator and denominator avoids division by zero, which aids in stratification of patients without PLNs into different prognostic groups (9). Recently, LODDS staging was thought a better predictor of survival in the small bowel neuroendocrine tumors compared with LNR and PLN staging (10). However, the actual prognostic value of LODDS in PanNENs remains unclear. Therefore, the aim of the current study was to determine the most appropriate nodal staging system for predicting cause-specific survival in PanNENs, by evaluating the prognostic ability of the PLN (or 8th edition AJCC N staging), LNR, and LODDS staging systems.

## Materials and Methods

The data of patients with PanNENs during the period 1988 to 2015 were obtained from the Surveillance, Epidemiology, and End Results (SEER) database. The following histological subtypes were included in the analysis, based on their International Classification of Diseases for Oncology, Third Edition codes: islet-cell adenocarcinoma (8150), malignant beta-cell tumor (8151), malignant alpha-cell tumor (8152), G-cell tumor (8153), mixed islet cell and exocrine tumor (8154), VIPoma (8155), malignant somatostatinoma (8156), carcinoid tumor (8240), enterochromaffin cell tumor(8241), goblet carcinoid tumor (8243), mixed adenoneuroendocrine carcinoma (8244), neuroendocrine carcinoid (8246), and atypical carcinoid tumor (8249).

The patient eligibility criteria were as follows: (1) histologically confirmed neuroendocrine tumors; (2) primary tumor located in the pancreas; (3) no other malignant tumors; (4) ≥18 years of age; (5) treatment by primary site surgery; (6) at least one lymph node was examined; (7) complete PLN data; (8) survival time of more than 0 months; and (9) close follow-up. In this study, the primary outcome was cause-specific survival, defined as death caused by PanNENs.

Scatterplots and Spearman’s correlation coefficients were used to evaluate the distributions of, and correlations among, continuous PLN, LNR, and LODDS variables. Smooth curves from restricted cubic spline were plotted to assess the non-linear relationship between three nodal staging systems and log hazard ratio (HR) by univariate Cox regression model (11). X-tile software (version 3.6.1; https://medicine.yale.edu/lab/rimm/research/software/) was used to determine the optimal cutoff points for the PLN, LNR, and LODDS staging systems. This software divides patients into two or three groups based on the χ2 value. Cross-validation was performed to derive corrected P-values (12). The discriminative efficacy of the different nodal staging systems was assessed by Harrell’s concordance index (C-index), which ranges from 0.5 (no predictive power) to 1 (complete differentiation) (13). Because the survival rate of the patients changed with time, we also used time-dependent area under the receiver operating characteristic curve (AUC) analysis to examine the discriminative abilities of the different nodal staging systems over a 10-year period (14). Univariate and multivariate analyses were performed to evaluate the prognostic performance of the three nodal staging systems and other possible prognostic factors. All factors with a P-value <0.2 in the univariate analysis were included in Cox proportional hazards multivariate models. Cases with missing data were excluded from the multivariate analysis.

All analyses were conducted using X-tile (version 3.6.1; Yale University School of Medicine, New Haven, CT, USA), SPSS (version 22.0; SPSS Inc., Chicago, IL, USA), and R software (version 3.5.3; R Foundation for Statistical Computing, Vienna, Austria). A P-value <0.05 was considered significant, and all tests were two-tailed.

## Results

### Characteristics of Patients

**Figure 1:**
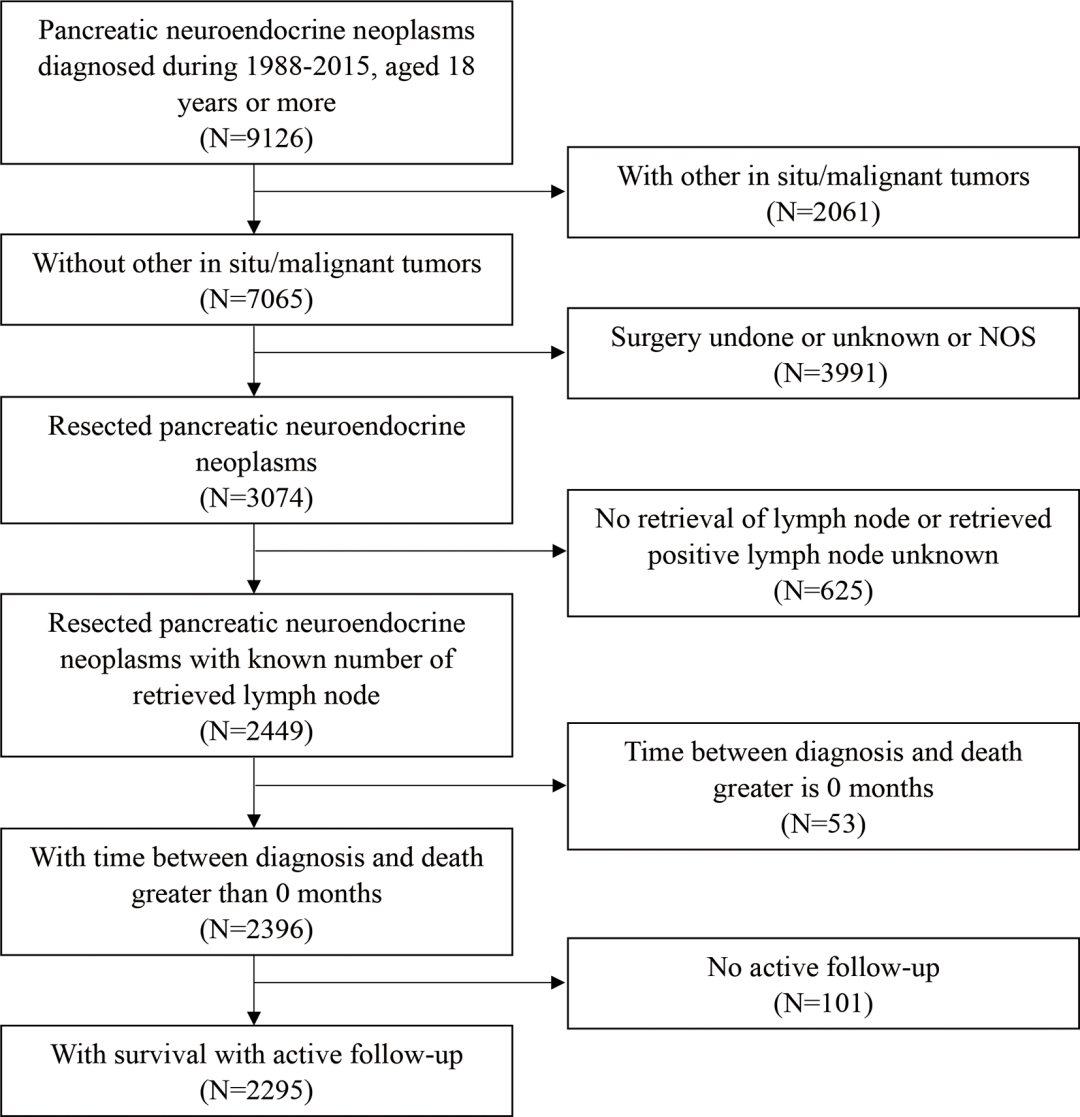
Flow diagram of patient selection.

A total of 2,295 patients with PanNENs who met the inclusion criteria were enrolled in this study (**Figure 1**); their clinical characteristics are described in **Table 1**. The study population was 78.7% white, 12.0% black, and 9.3% other races. About 53% of the patients were male and 47% were female. The median age at diagnosis was 57 years. About 90.0% of patients underwent a local or partial pancreatectomy. Most tumors were located in the tail of the pancreas (40.2%) or the head of the pancreas (34.4%). The vast majority of tumors were non-functioning (97.4%) and well-to moderately differentiated on histological examination (91.6%). The results of the univariate analysis showed that age, sex, marriage, extent of resection, histologic grade, T staging, M staging, and the number of examined lymph nodes had significant prognostic value (P < 0.05).

**Table 1:** Clinicopathologic characteristics of patients.

Characteristic		Patients No. (%)	Univariate Analysis	
			HR (95% CI)	P
Age (years)	≤60	1,370 (59.7)	1 (ref)	**<0.001**
	>60	925 (40.3)	1.700 (1.414–2.045)	
Sex	Female	1,075 (46.8)	1 (ref)	**0.032**
	Male	1,220 (53.2)	1.224 (1.017–1.473)	
Marriage	Yes	1,462 (66.9)	1 (ref)	**0.007**
	No	724 (33.1)	1.303 (1.075–1.580)	
Race	White	1,793 (78.7)	1 (ref)	0.402
	Black	273 (12.0)	0.928 (0.691–1.247)	
	Others	211 (9.3)	0.798 (0.562–1.134)	
Tumor location	Head	738 (34.4)	1 (ref)	0.164
	Body	301 (14.0)	0.809 (0.584–1.122)	
	Tail	862 (40.2)	0.818 (0.654–1.023)	
	Others	245 (11.4)	1.058 (0.791–1.416)	
Extent of resection	Local or partial pancreatectomy	2,035 (90.0)	1 (ref)	**<0.001**
	Total pancreatectomy	226 (10.0)	1.664 (1.289–2.147)	
Functionality	Nonfunctioning	2,236 (97.4)	1 (ref)	0.207
	Functioning	59 (2.6)	0.701 (0.403–1.218)	
Histologic grade	Well differentiated or moderately differentiated	1,728 (91.6)	1 (ref)	**<0.001**
	Poorly differentiated or undifferentiated	159 (8.4)	5.221 (4.081–6.679)	
T staging^a^	T1	497 (28.3)	1 (ref)	**<0.001**
	T2	530 (30.2)	1.886 (1.197–2.971)	
	T3	475 (27.1)	3.370 (2.198–5.165)	
	T4	254 (14.5)	6.247 (4.066–9.596)	
M staging	M0	1,340 (87.3)	1 (ref)	**<0.001**
	M1	195 (12.7)	3.355 (2.322–4.847)	
Number of examined lymph nodes^b^, median (IQR)		10 (12)	1.012 (1.002–1.023)	**0.016**

a T staging was graded according to the 8th edition AJCC staging system for well-differentiated PanNETs.

b Continuous variable.

No., number of patients; HR, hazard ratio; IQR, interquartile range.

All variables with a P-value <0.2 in the univariate analysis were then included in multivariate analysis. The bold values means that the P value is less than 0.05. The bold values means that the P value is less than 0.05.

### Characteristics of Three Nodal Staging Schemes

**Figure 2:**
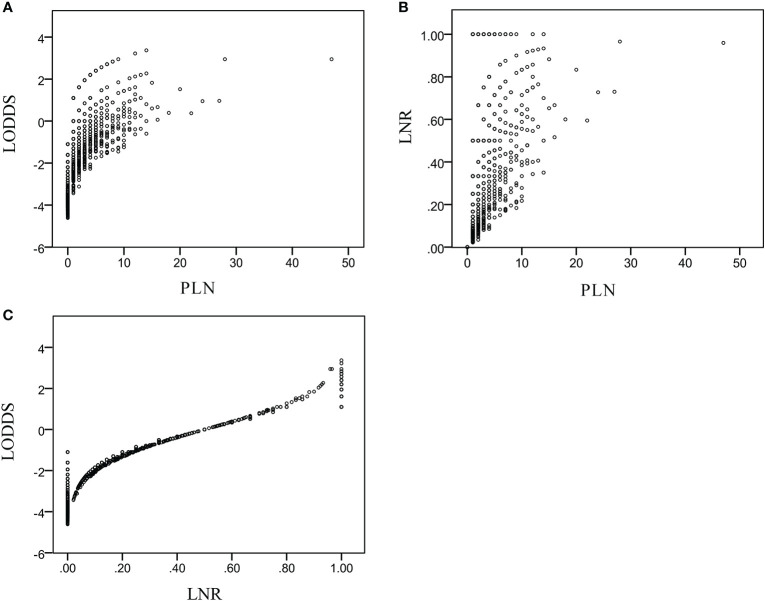
Distribution characteristics of LODDS and PLN **(A)**, LNR and PLN **(B)**, LODDS and LNR **(C)**.

Scatterplots (**Figure 2**) were created to assess the relationships of PLN, LNR, and LODDS data. Each PLN value could be divided into different LNR and LODDS values. Each LODDS value had a one-to-one correspondence with an LNR value, except when LNR equaled 0 or 1. The LODDS data were more strongly correlated with the LNR than PLN data (r = 0.752 vs. r = 0.703).

**Figure 3:**
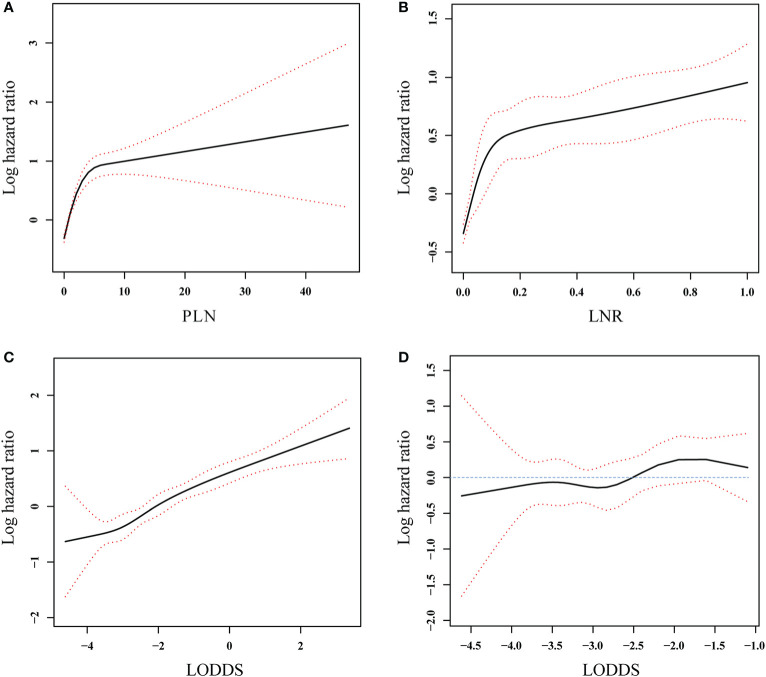
Association between PLN **(A)**, LNR **(B)**, and LODDS **(C)** and log hazard ratio in all patients and association between LODDS **(D)** and log hazard ratio in the patients without positive lymph nodes involvement. The solid line and the red dashed lines represent the estimated log hazard ratio and the 95% confidence interval, respectively.

Restricted cubic splines (**Figure 3**) were used to assess the relationships between the three lymph node staging systems and log HR. A nonlinear association was found, and the mortality risk increased as the LODDS, LNR, and PLN values increased in all patients with PanNENs. **Figure 3D** showed that the confidence interval (CI) included 0, suggesting that LODDS was neither a risk factor nor a protective factor in node-negative patients.

### Three-Category Nodal Staging Schemes

To compare the three lymph node staging schemes from three-category perspective, the continuous variables of the PLN, LNR, and LODDS were divided into three groups using X-Tile software. The PLN staging system includes three groups: PLN1(= 0), n = 1,425 (62.1%); PLN2 (1–3), n = 570 (24.8%); and PLN3 (≥4), n = 300 (13.1%). It happens to coincide with the 8th edition AJCC N staging for the PanNECs. The LNR also yielded three risk groups: LNR1 (= 0), n = 1, 425 (62.1%); LNR2 (0–0.28), n = 474 (20.7%); and LNR3 (>0.28), n = 396 (17.3%). Finally, the LODDS staging consisted of the following three groups: LODDS1 (≤−2.56), n = 961 (41.9%); LODDS2 (−2.56 to −0.66), n = 968 (42.2%); and LODDS3 (>−0.66), n = 366 (15.9%) (**Table 2**).

**Table 2:** Univariate and multivariate analysis of three-category nodal staging schemes.

Characteristic		Patients No. (%)	Univariate		Multivariate	
			HR (95% CI)	P	HR (95% CI)	P^a^
PLN staging	PLN1 (=0)	1,425 (62.1)	1 (ref)	**<0.001**	1 (ref)	**0.015**
	PLN2 (1 to 3)	570 (24.8)	1.767 (1.429–2.184)		1.654 (0.895–3.060)	
	PLN3 (≥4)	300 (13.1)	2.752 (2.177–3.478)		2.559 (1.361–4.810)	
LNR staging	LNR1 (=0)	1,425 (62.1)	1 (ref)	**<0.001**	1 (ref)	**0.016**
	LNR2 (0 to 0.28)	474 (20.7)	1.743 (1.384–2.196)		2.414 (1.344–4.336)	
	LNR3 (>0.28)	396 (17.3)	2.438 (1.968–3.022)		1.592 (0.831–3.049)	
LODDS staging	LODDS1 (≤−2.56)	961 (41.9)	1 (ref)	**<0.001**	–	0.093
	LODDS2 (−2.56 to −0.66)	968 (42.2)	1.471 (1.168–1.852)			
	LODDS3 (>−0.66)	366 (15.9)	2.532 (1.978–3.242)			

a Adjusted for age, sex, marriage, tumor location, extent of resection, histological grade, T staging, M staging, and the number of examined lymph nodes.

No., number of patients; HR, hazard ratio. The bold values means that the P value is less than 0.05.

The results of the univariate analysis showed that the three-category PLN, LNR, and LODDS staging systems all had significant prognostic value. However, in the multivariate analysis, only PLN and LNR staging were independent prognostic factors of a worse survival (**Table 2**).

**Figure 4:**
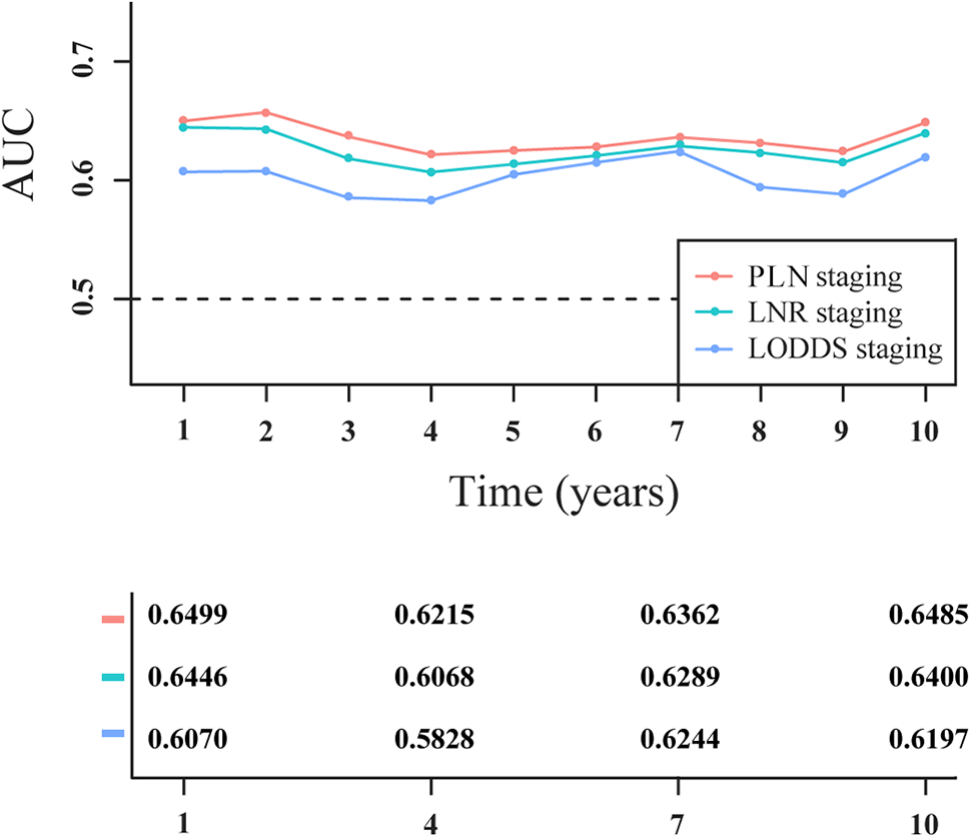
Time-dependent area under the receiver operating characteristic curve (AUC) in patients with PanNENs for three-category staging schemes.

Based on the C-index value, PLN staging (0.642; 95% CI: 0.611–0.673) had higher prognostic value compared with LNR staging (0.636; 95% CI: 0.606–0.666) and LODDS staging (0.617; 95% CI: 0.585–0.648). As shown in **Figure 4**, PLN staging consistently had higher AUC values at the 1-, 2-, 3-, 4-, 5-, 6-, 7-, 8-, 9-, and 10-year follow-ups than LNR or LODDS staging in the survival analysis, suggesting that it was better able to distinguish between the prognosis of patients with PanNENs, consistent with the C-index result.

As shown in **Table 3**, we then compared the survival rates of patients staged using the PLN, LNR, and LODDS staging systems according to the number of examined lymph nodes. For patients in each PLN staging subgroup (PLN1, PLN2, and PLN3), the survival was highly homogenous among different groups of examined lymph nodes (all P^a^ > 0.05). For patients in LNR3, LODDS2, and LODDS3 subgroups, statistical differences in the survival could be observed among different groups of examined lymph nodes $\left({\text{P}}_{\text{LNR}3}^{\text{a}}=\;0.035,\;{\text{P}}_{\text{LODDS}2}^{\text{a}}=0.001,\;{\text{P}}_{\text{LODDS}3}^{\text{a}}=\;0.025\right)$. To find out why LNR and LODDS systems, rather than PLN, showed intra-group heterogeneity, the prognoses of patients with 1–5, 6–10, 11–15, and 16–20 examined lymph nodes were compared with those with >20 examined lymph nodes respectively. In each PLN staging subgroup (PLN1, PLN2, and PLN3), the prognoses of patients with ≤ 20 examined lymph nodes were similar to those of patients with >20 examined lymph nodes (all p^b^ > 0.05), suggesting that an insufficient number of examined lymph nodes would not lead to PLN staging migration. By contrast, the ratio lymph nodal staging schemes (LNR and LODDS) overestimated the HR for patients with insufficient number of examined lymph nodes. For example, when the number of examined lymph nodes was between 1 and 5, patients were assigned only to the LODDS2 and LODDS3 subgroups, and not to LODDS1. However, patients with 1–5 examined lymph nodes had a significantly better prognosis than those with >20 examined lymph nodes in the LODDS2 and LODDS3 subgroups (both P^b^ < 0.001).

**Table 3:** Survival analysis on the basis of three-category nodal staging schemes according to the number of examined lymph nodes (ELNs).

	1–5 ELNs			6–10 ELNs			11–15 ELNs			16–20 ELNs			>20 ELNs		P^a^
	No.	Time	P^b^	No.	Time	P^b^	No.	Time	P^b^	No.	Time	P^b^	No.	Time	
**PLN staging**															
PLN1	512	169.4	0.424	356	168.6	0.650	249	163.2	0.782	149	166.9	0.731	159	136.2	0.371
PLN2	172	125.4	0.660	128	146.6	0.822	105	161.8	0.385	76	93.9	0.943	89	129.2	0.689
PLN3	17	116.3	0.148	63	122.7	0.068	75	115.4	0.257	54	103.3	0.130	91	93.9	0.271
**LNR staging**															
LNR1	512	169.4	0.424	356	168.6	0.650	249	163.2	0.782	149	166.9	0.731	159	136.2	0.371
LNR2	43	128.2	0.307	87	161.9	**0.019**	110	161.7	**0.047**	90	101.7	0.449	144	133.8	0.101
LNR3	146	121.9	**0.001**	104	117.5	**0.022**	70	113.1	0.143	40	105.5	0.076	36	59.8	**0.035**
**LODDS staging**															
LODDS1	–	–	–	356	168.6	0.102	249	163.2	0.462	156	166.3	0.290	200	131.3	0.470
LODDS2	555	166.9	**<0.001**	101	157.5	**0.016**	117	154.4	0.065	87	102.2	0.377	108	129.4	**0.001**
LODDS3	146	121.9	**<0.001**	90	116.5	**0.023**	63	118.7	0.094	36	104.0	0.053	31	57.1	**0.025**

a Comparison of survival among different groups of examined lymph nodes.

b Comparison of survival with group of >20 examined lymph nodes.

No., number of patients; Time, mean cause–specific survival (months). The bold values means that the P value is less than 0.05.

**Figure 5:**
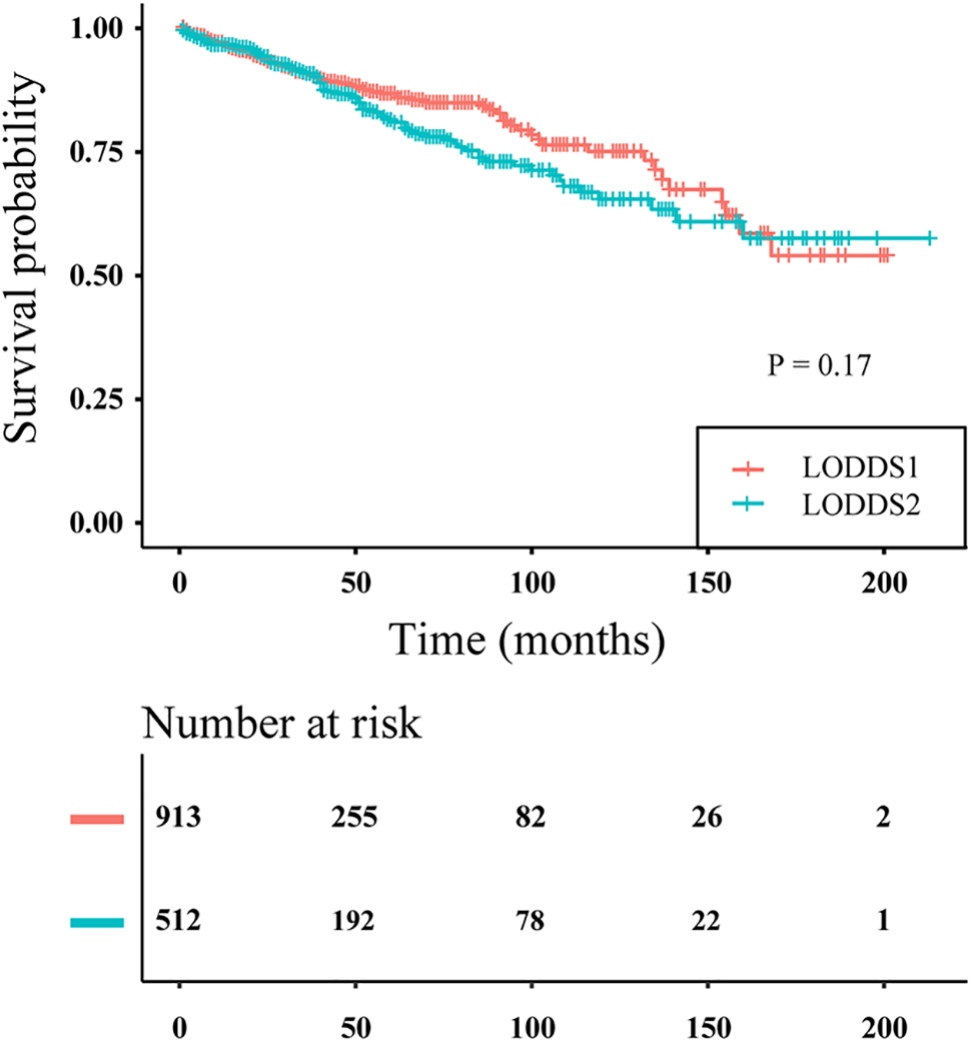
Disease-specific survival curves of PanNENs patients without positive lymph nodes according to three-category LODDS staging (log rank P = 0.17).

LODDS was said to have an inherent advantage for assessing node-negative patients. However, in our study, node-negative patients were assigned only to LODDS1 and LODDS2, and not to LODDS3. The survival curves of node-negative patients stratified by LODDS staging are plotted in **Figure 5**. No significant difference was observed in survival probability between the LODDS1 and LODDS2 subgroups (P > 0.05), indicating that three-category LODDS staging could not stratify node-negative patients into different risk groups.

### Two-Category Nodal Staging Schemes

We compared the different lymph node staging schemes from the perspective of two categories. Using X-Tile software, two-stage solutions were derived for the PLN, LNR, and LODDS staging systems, as follows. For the PLN staging system, PLN1 (= 0), n = 1,425 (62.1%); and PLN2 (>0), n = 870 (37.9%), consistent with the 8th edition AJCC N staging system for the neuroendocrine pancreas. For the LNR staging system, LNR1 (= 0), n = 1,425 (62.1%); and LNR2 (>0), n = 870 (37.9%). For the LODDS staging system, LODDS1 (≤ −0.93), n = 1,869 (81.4%); and LODDS2 (>−0.93), n = 426 (18.6%) (**Table 4**). Only the PLN and LODDS staging systems are discussed in the following section, as the LNR and PLN systems were not different. The result was similar to that of three-category staging schemes.

**Table 4:** Univariate and multivariate analysis of two-category nodal staging schemes.

Characteristic		Patients No. (%)	Univariate		Multivariate	
			HR (95% CI)	P	HR (95% CI)	P^a^
PLN staging	PLN0 (=0)	1,425 (62.1)	1 (ref)	**<0.001**	1 (ref)	**0.009**
	PLN1 (>0)	870 (37.9)	2.076 (1.722–2.502)		1.999 (1.190–3.359)	
LNR staging	LNR1 (=0)	1,425 (62.1)	1 (ref)	**<0.001**	1 (ref)	**0.009**
	LNR2 (>0)	870 (37.9)	2.076 (1.722–2.502)		1.999 (1.190–3.359)	
LODDS staging	LODDS1 (≤−0.93)	1,869 (81.4)	1 (ref)	**<0.001**	–	0.317
	LODDS2 (>−0.93)	426 (18.6)	1.984 (1.636–2.405)			

a Adjusted for age, sex, marriage, tumor location, extent of resection, histological grade, T staging, M staging, and the number of examined lymph nodes.

No., number of patients; HR, hazard ratio. The bold values means that the P value is less than 0.05.

The two-category PLN and LODDS staging were significantly correlated with prognosis in the univariate analysis. However, only the two-category PLN staging was an independent prognostic factor in the multivariate analysis (**Table 4**).

**Figure 6:**
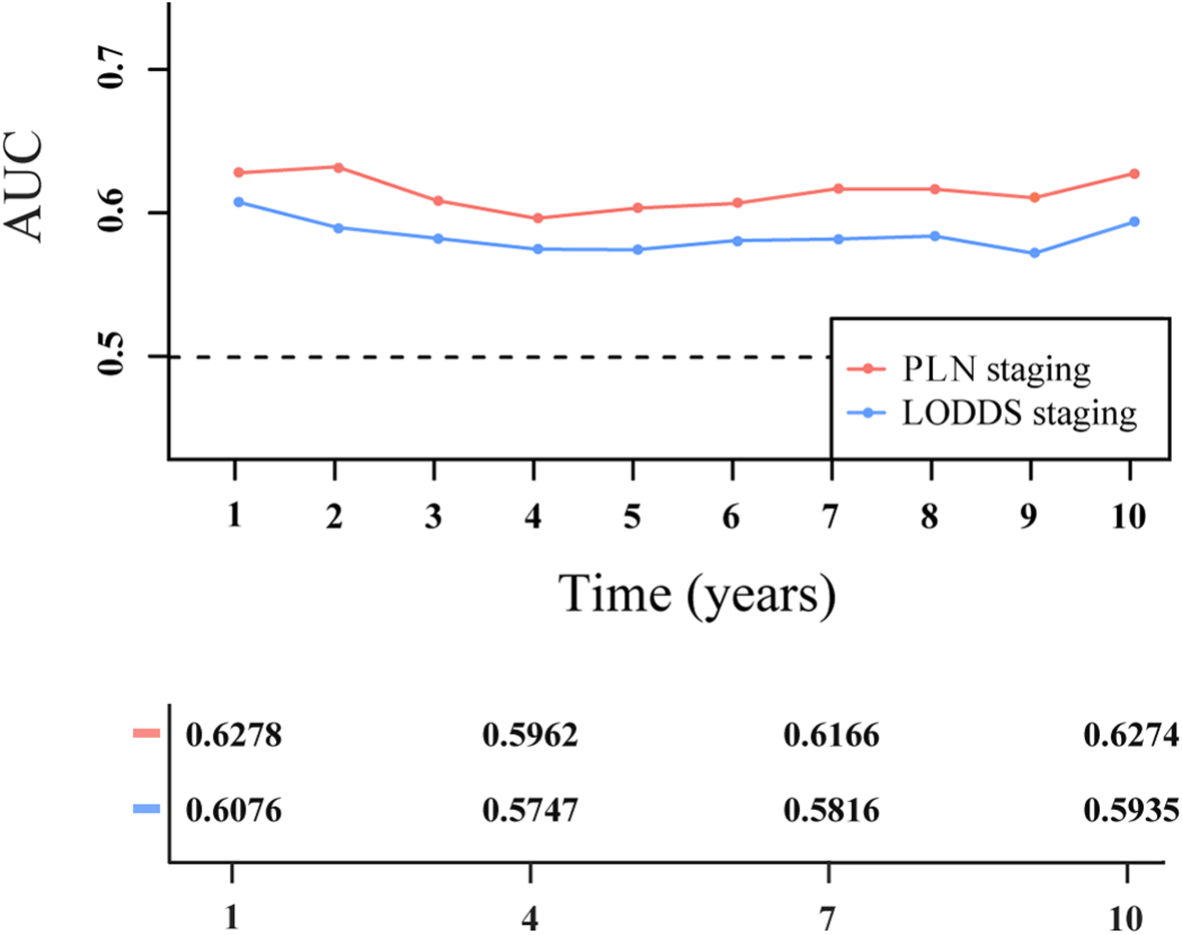
Time-dependent area under the receiver operating characteristic curve (AUC) for two-category staging schemes in patients with PanNENs.

As measured by Harrell’s C-index, PLN staging (0.623; 95% CI: 0.595–0.651) had better discriminatory capacity than LODDS staging (0.591; 95% CI: 0.564–0.619). As shown in **Figure 6**, PLN staging had higher 1-, 2-, 3-, 4-, 5-, 6-, 7-, 8-, 9-, and 10-year AUC values than LODDS staging.

We compared the survival rates of patients using the PLN and LODDS staging systems according to the number of examined lymph nodes. As shown in **Table 5**, for patients in each PLN staging subgroup (PLN1 and PLN2), the survival was highly homogenous among different groups of examined lymph nodes $\left({\text{P}}_{\text{PLN}1}^{\text{a}}=\;0.371,\;{\text{P}}_{\text{PLN}2}^{\text{a}}=0.249\right)$. However, significant differences in survival were observed in the LODDS1 and LODDS2 subgroups $\left({\text{P}}_{\text{LODDS}1}^{\text{a}}=\;0.013,\;{\text{P}}_{\text{LODDS}2}^{\text{a}}=0.008\right)$, indicating that LODDS staging had increased intra-group heterogeneity compared with PLN staging.

**Table 5:** Survival analysis on the basis of two-category nodal staging schemes according to the number of examined lymph nodes (ELNs).

	1–5 ELNs			6–10 ELNs			11–15 ELNs			16–20 ELNs			>20 ELNs		P^a^
	No.	Time	P^b^	No.	Time	P^b^	No.	Time	P^b^	No.	Time	P^b^	No.	Time	
**PLN staging**															
PLN1	512	169.4	0.424	356	168.6	0.650	249	163.1	0.782	149	166.9	0.731	159	136.2	0.371
PLN2	189	125.6	0.070	191	143.0	**0.042**	180	138.7	0.141	130	104.7	0.170	180	125.4	0.249
**LODDS staging**															
LODDS1	532	168.2	**0.018**	443	172.3	**<0.001**	356	170.6	**0.020**	237	143.7	0.176	301	154.5	**0.013**
LODDS2	169	125.4	**<0.001**	104	117.5	**0.013**	73	112.4	0.101	42	106.9	**0.039**	38	58.3	**0.008**

a Comparison of survival among different groups of examined lymph nodes.

b Comparison of survival with group of >20 examined lymph nodes.

No., number of patients; Time, mean cause–specific survival (months). The bold values means that the P value is less than 0.05.

Patients were assigned only to the LODDS1 subgroup when the number of PLNs was zero. Therefore, the two-category LODDS staging failed to stratify node-negative patients into distinct survival groups.

## Discussion

Lymph node metastasis was thought to be an independent predictor of poor outcomes in patients with PanNENs (15, 16). AJCC staging based on the number of PLNs is the most widely accepted nodal staging system. The staging algorithm for PanNENs is the same as that for pancreatic carcinomas in the 7th edition AJCC cancer staging manual (17). A large retrospective cohort study reported that European Neuroendocrine Tumor Society (ENETS) system, which was specifically designed for staging PanNENs, better distinguishes among stages and has higher predictive ability than the 7th edition AJCC system (18, 19). Therefore, the 8th edition AJCC staging was modified as follows: well-differentiated PanNETs are now staged using the staging system for the neuroendocrine pancreas (which is consistent with the ENETS system), while poorly differentiated PanNECs are still staged using the system for pancreatic carcinomas. The 8th edition AJCC N staging for PanNETs is a two-category system (N0: no metastasis; N1: ≥1 metastatic lymph nodes); the 8th edition AJCC N staging for PanNECs is a three-category system (N0: no metastasis; N1: 1–3 metastatic lymph nodes; N2: ≥4 metastatic lymph nodes) (9). Based on the number of PLNs, we used X-tile software to divide the PanNENs patients in the SEER database into two populations (0, and >0), and then into three populations (0, 1–3, and ≥4). It was interesting to note that these divisions were consistent with the AJCC N staging system adopted for PanNETs and PanNECs, indicating that the cut-off points in the current 8th edition AJCC N staging system are reasonable. In addition, our study demonstrated that three-category nodal staging had better discriminatory capacity than the two-category system to predict cause-specific survival of PanNENs, as measured by the C-index and time-independent AUC. This conclusion is similar to that of a previous small retrospective study, which proposed that three-category nodal staging is accurate for predicting the recurrence of both PanNETs and PanNECs (5).

The PLN staging (or 8th edition AJCC N staging) depends only on the number of PLNs; the impact of the number of examined lymph nodes (or negative lymph nodes) is not considered. When the number of PLNs is the same, patients with an insufficient number of examined lymph nodes might have a poorer prognosis; this phenomenon of understaging is called staging migration or Will Rogers phenomenon (20).

The LNR staging system may reduce the risk of the staging migration in some cancers, such as gastric cancers (21). However, the utility of the LNR is limited when the ratio value is zero. Another drawback is that patients with the same LNR count (e.g., 1/1 vs. 30/30) may have different prognoses. The optimal LNR cutoff in PanNENs is controversial and varies among studies, perhaps due to differences in the end points or number of patients. Two small retrospective studies assessed the LNR using cutoffs of 0.2 and 0.07, and found that it was a significant predictor of recurrence in patients with PanNENs (22, 23). Using SEER data, Liu et al. showed that an LNR > 0.40 was a significant adverse prognostic factor with respect to overall survival (24). Gaitanidis et al. reported that an LNR ≥ 0.5 was independently associated with a worse cause-specific survival and the LNR-based staging system was superior to 8th edition AJCC N staging (8). In our study, which contained the largest number of PanNENs patients among all of these studies, LNR staging predicted cause-specific survival in patients with PanNENs, but did not show higher prognostic utility than the AJCC N staging system.

LODDS is a new approach to evaluate nodal status. LODDS comprehensively considers the numbers of PLNs and negative lymph nodes, and thus can also reduce the likelihood of staging migration due to examination of an insufficient number of lymph nodes. In addition, it overcomes the major shortcoming of the LNR: the LNR value does not increase with an increase in the number of PLNs when all examined lymph nodes are positive. Besides, the LODDS value decreases with an increase in the number of negative lymph nodes when all examined lymph nodes are negative. Therefore, LODDS has a unique value for risk stratification of the node-negative patients, which LNR and PLN staging systems do not possess (25). However, the practical utility of LODDS remains controversial. For example, Morales-Oyarvide et al. found no obvious improvement in prognostic value when using the LNR and LODDS classifications compared with the PLN classification in pancreatic ductal adenocarcinomas (25). To the best of our knowledge, the prognostic role of LODDS has never been analyzed in PanNENs. Our research demonstrates that LODDS fails to discriminate node-negative patients in terms of survival. In the two-category nodal staging, the node-negative patients were not reclassified into different LODDS subgroups; they were all assigned to the LODDS1 subgroup. In the three-category nodal staging, the node-negative patients could be reclassified LODDS2 and LODDS3 subgroups, but the difference in survival between them was not significant. In addition, restricted cubic spline functions indicate that LODDS is neither a risk factor nor a protective factor when the number of PLNs is zero.

The current study demonstrates that PLN staging performs better than LNR and LODDS staging for predicting cause-specific survival in patients with PanNENs after pancreatic resection surgery from both two-category and three-category perspective. Firstly, PLN and LNR staging, but not LODDS staging, were independent prognostic factors in the multivariate analysis. Secondly, PLN staging showed greater C-index and AUC values than the LNR and LODDS staging systems, corresponding to better discriminatory capacity. Thirdly, and most importantly, no significant differences in survival were observed among patients in the same PLN staging subgroup according to the examined lymph node value (high or low). On the contrary, LNR and LODDS systems showed intra-group heterogeneity due to overestimating the risk of an insufficient number of examined lymph nodes. In consideration of the relatively good prognosis and limited prognostic value of lymph node metastasis in the patients with PanNENs, the ratio lymph nodal staging schemes (LNR and LODDS) designed to reduce intra-group heterogeneity was actually counterproductive.

The current study had some inherent limitations associated with the inherent limitations of the SEER database. For example, the SEER database does not include several important prognostic factors, such as patients’ symptoms, margin status, and chemotherapy, which may have affected the results of our Cox multivariate analysis. In addition, we excluded patients with missing data on the number of positive lymph nodes and examined lymph nodes, which may have caused selection bias. However, these are common shortcomings of retrospective and population-based studies. Given the rarity of PanNENs, retrospective studies based on large cancer registries are still of great significance, and prospective studies are needed to validate our conclusions.

In conclusion, the predictive value of the PLN staging system was not affected by an insufficient number of examined lymph nodes, while use of the LNR and LODDS systems was associated with an increased risk of nodal staging migration. The LODDS system did not stratify node-negative patients in terms of risk. Currently, the PLN staging (or 8th edition AJCC N staging) is superior to the LNR and LODDS staging.

## Data Availability Statement

Publicly available datasets were analyzed in this study. This data can be found here: https://seer.cancer.gov/data/.

## Author Contributions

BG and DZ conceived the study. BG, ZL, XQ, WZ, and YJ collected and analyzed data. BG, DZ, and ZL wrote the manuscript. WW revised it critically. All authors contributed to the article and approved the submitted version.

## Funding

This work was supported by the National Natural Science Foundation of China (no. 81572307 and 81773096) and the Major Project of Medical and Health Technology Development Program in Zhejiang Province (No. 7211902).

## Conflict of Interest

The authors declare that the research was conducted in the absence of any commercial or financial relationships that could be construed as a potential conflict of interest.
